# Magnetoencephalographic brain activity evoked by the optic-flow task is correlated with β-amyloid burden and parahippocampal atrophy

**DOI:** 10.1016/j.nicl.2024.103700

**Published:** 2024-11-04

**Authors:** Shoko Tsuchimine, Kiwamu Kudo, Junji Komatsu, Shutaro Shibata, Sachiko Kitagawa, Yoshihiro Misaka, Moeko Noguchi-Shinohara, Kenjiro Ono, Hirofumi Morise, Takashi Asakawa

**Affiliations:** a Medical Imaging Business Center, Ricoh Company, Ltd., Kanazawa, Japan; b Department of Neurology and Neurobiology of Aging, Kanazawa University, Kanazawa, Japan; c Department of Preemptive Medicine of Dementia, Kanazawa University, Kanazawa, Japan

**Keywords:** Alzheimer’s disease, Magnetoencephalography, Optic-flow task, Visuospatial impairment, Superior parietal lobule, Parahippocampal gyrus volume

## Abstract

Visuospatial perception is often impaired in people with Alzheimer’s disease (AD). Because visuospatial information is thought to be processed in the visual dorsal stream, it is believed that brain activities in the dorsal stream will be altered in AD patients. In this study, we investigated whether regional brain activity related to visuospatial perception were associated with AD progression markers. An optic-flow task, which activates the dorsal stream associated with visuospatial perception, was performed, and the brain activities evoked by the task were evaluated using magnetoencephalography (MEG). First, we evaluated the responses to optic-flow stimuli in 21 cognitively unimpaired participants and determined the regions of interest (ROIs) where optic-flow activities were activated. Task-related activations were observed in 14 cortical regions including the dorsal stream: the right and left medial ventral occipital cortex (MVOcC), lateral occipital cortex (LOcC), precuneus (Pcun), inferior parietal lobule (IPL), superior parietal lobule (SPL), posterior superior temporal sulcus (pSTS), and fusiform gyri (FuG). Next, we performed correlation analyses between task-related activity in each ROI and two AD progression markers, global amyloid burden and parahippocampal gyrus (PHG) volume, for 25 participants who underwent amyloid positron emission tomography (PET) scans. We found that the global amyloid burden was negatively correlated with task-related activity in the left MVOcC and right SPL [*r* = -0.488 (*p* = 0.013) and *r* = -0.421 (*p* = 0.038), respectively]. Furthermore, significant positive correlations were observed between PHG volume and task-related activity in both the left and right SPL [*r* = 0.500 (*p* = 0.011) and *r* = 0.549 (*p* = 0.005), respectively]. Since the SPL is known to be responsible for visuospatial perception, these results suggest that MEG neuronal activity of patients performing the optic-flow activity can detect changes in brain activity associated with visuospatial impairment related to AD.

## Highlights

• Visuospatial perception is impaired in AD, affecting brain activity in the visual dorsal stream.
• Brain activities in the SPL related to visuospatial perception during optic-flow are correlated with global amyloid burden and PHG volume in AD patients.
• The MEG of patients performing the optic-flow might capture changes in brain activity associated with visuospatial impairment caused by AD.

## 1 Introduction

Memory and cognitive functions are commonly impaired in Alzheimer’s disease (AD), but visuospatial functions are also often impaired even in the early stages of AD (Albers et al., 2015, Cronin-Golomb et al., 1995). Visuospatial impairments include getting lost in familiar environments, forgetting locations of personal items, difficulty driving or parking a car, etc. Moreover, deficits in reading, numeric operations, and orientation are caused by visuospatial impairment and do not necessarily stem from the impairments in memory or language commonly observed in AD (Nguyen et al., 2003). Therefore, it may be useful to evaluate visuospatial deficits in AD patients, which could support diagnosis and enable new intervention approaches, and also allows researchers to track disease progression and explore the disrupted neural networks underlying dementia symptoms (Basu et al., 2023).

Optic flow is the displacement of retinal images of objects moving coherently as a whole. Optic flow enables an individual to make inferences about his or her own motion direction relative to the environment and surrounding objects. Optic-flow tasks have been used to study visuospatial orientation capacities. Several behavioral studies using optic-flow tasks with radially looming dots have shown that patients with mild cognitive impairment (MCI) and AD dementia have difficulty in perceiving optic-flow stimuli (Kavcic et al., 2011, Monacelli et al., 2003, Tetewsky and Duffy, 1999). Compared to healthy elderly individuals, AD patients are worse at perceiving self-motion induced by optic-flow stimuli (Mapstone et al., 2006), which results in higher thresholds for the perception of these stimuli (Kavcic and Duffy, 2003). However, it is still unclear which brain regions are involved in the inability to perceive optic-flow stimuli in AD patients.

Previous neuroimaging studies of healthy volunteers using functional magnetic resonance imaging (fMRI) and magnetoencephalography (MEG) have revealed that optic-flow stimuli activate the areas in the dorsal stream, which is composed of the primary visual cortex (V1), fifth visual cortex (V5/hMT + ), precuneus (Pcun), superior parietal lobule (SPL), and inferior parietal lobule (IPL) (Cardin and Smith, 2010, Livingstone and Hubel, 1988, Pitzalis et al., 2015, Smith et al., 2006, Uesaki and Ashida, 2015). These regions have also been shown to be related to neural basis of visuospatial impairment in AD patients, which has been demonstrated by previous studies using visuospatial tasks other than the optic-flow task (Thulborn et al., 2000). Thulborn et al. (Thulborn et al., 2000) and Prvulovic et al. (Prvulovic et al., 2002) demonstrated that there was less activity in the SPL in AD patients than in controls using a visuospatial guided saccade paradigm and a visuospatial angle discrimination task, respectively. Using another visuospatial task to estimate the perception of an object’s depth and motion, Thiyagesh et al. showed that AD patients exhibited hypoactivation in the V5, SPL, parietooccipital cortex, and premotor cortices, as well as greater compensatory activation in the IPL (Thiyagesh et al., 2009). Moreover, Hof (Hof, 1994) reported that impairment in projections from V1 to the middle temporal (MT) area causes deficits in higher-order visual processing in AD patients. Overall, the brain regions in the dorsal stream are involved in visuospatial processing, and the brain activities in those areas decrease in AD patients. We therefore expect that the brain activities in the dorsal stream evoked by the optic-flow task would also be altered in AD patients. Clearly testing these expectations requires measurements of task-related responses of brain activity with high temporal resolution because visual responses usually occur approximately 100 ms after a visual stimulus (Kavcic et al., 2006, Noguchi-Shinohara et al., 2021, Yamasaki and Tobimatsu, 2018). MEG allows us to measure brain processing during the optic-flow task in milliseconds, which may capture the transition of millisecond-order responses related to visual spatial processing from V1 to the parietal lobe. This study used MEG to investigate whether the regional brain activity induced by the optic-flow task is associated with AD. To investigate these associations, we analyzed the correlations between the task-related activity and AD progression markers.

We used two AD progression markers: global amyloid burden and parahippocampal gyrus (PHG) volume. The aggregation of beta-amyloid (Aβ) peptides and formation of Aβ plaques are pathological hallmarks of preclinical and clinical AD (Jack et al., 2018, Long and Holtzman, 2019, McKhann et al., 2011). The brain regions associated with Aβ accumulation in AD patients include the association cortices, including the prefrontal, orbitofrontal, parietal, temporal, and cingulate cortices, and the precuneus. Furthermore, a substantial amount of fibrillar forms of Aβ aggregation in the occipital lobes has also been reported (Bailly et al., 2015, Barthel et al., 2011, Jack et al., 2008, Klunk et al., 2004). Another marker of AD is parahippocampal atrophy; previous histological studies have shown that the earliest neuropathological changes in AD occur in the entorhinal cortex, which is the anterior part of the PHG (Braak and Braak, 1990, Van Hoesen, 1982). Several MRI studies have also suggested that volume measurements of the entorhinal cortex provide greater sensitivity than volume measurements of the hippocampus when detecting early-stage AD (deToledo-Morrell et al., 2004, Dickerson et al., 2001, Pennanen et al., 2004). In two comprehensive reviews (Leandrou et al., 2018, Zhou et al., 2016), the authors concluded that structural changes in the early stages of the disease are more pronounced in the entorhinal cortex. Furthermore, this area has been ascribed central roles in visuospatial processes (Epstein et al., 2003, Park and Chun, 2009, Rajimehr et al., 2011). We therefore hypothesized that the optic-flow task related to visuospatial cognition might be correlated with PHG volume.

In the present study, we investigated the association of the regional brain activity induced by the optic-flow task with two AD markers, global amyloid burden and PHG volume, using MEG. We hypothesized that regional neural activities induced by optic-flow task are related to AD markers, which would explain the impairment of visuospatial perception associated with AD. All participants (n = 45) in this study underwent the MEG scan with the optic-flow task. In this paper, we first define normative task-related neural activity using 21 healthy elderly individuals among them, and then we assess the association of regional task-related brain activity with AD pathology using the data of 25 participants who underwent PET scan as well as the MEG scan.

## 2 Methods

### 2.1 Participants

This study included 24 patients who met the criteria of the National Institute of Aging–Alzheimer’s Association (NIA-AA) (Albert et al., 2011, Jack et al., 2018) for Alzheimer’s disease or MCI due to AD and 21 cognitively unimpaired (CU) older adults. All participants were recruited from among community-dwelling individuals or outpatients at Kanazawa University Hospital. Twenty-one CU participants were included in the analyses to elucidate typical brain responses to optic-flow stimuli. Twenty-five of the 45 participants underwent amyloid-PET scans (1 CU participant, 16 MCI patients, and 8 AD patients). These 25 participants were included in the correlation analyses to investigate the association of task-related brain activities with two markers of AD, global amyloid burden and PHG volume.

All patients were diagnosed by neurologists. The patients were not taking medications that act on the central nervous system (i.e., cholinesterase inhibitors, N-methyl-d-aspartate receptor antagonists, antipsychotics, anticholinergics, or antidepressants). The CU participants also had no history of psychiatric or neurological diseases and were not taking medications acting on their central nervous system. There was no difference in the visual acuity of the participants, and there were not the participants who complained about difficulty in seeing the optic-flow task due to presbyopia. Also, none of the participants had ophthalmological diseases. The cognitive profiles of participants were evaluated using the Mini-Mental State Examination (MMSE) (Folstein et al., 1975). A structured caregiver interview was used to assess the Clinical Dementia Rating Scale (CDR) score of each participant. This study was carried out according to the Declaration of Helsinki guidelines, and all procedures involving human subjects were approved by the Ricoh Ethics Review Board (IRB number: 2019–01-03) and Kanazawa University Medical Ethics Review Board (IRB number: 3041). Written informed consent was obtained from all participants.

### 2.2 Optic-flow task

**Fig. 1:**
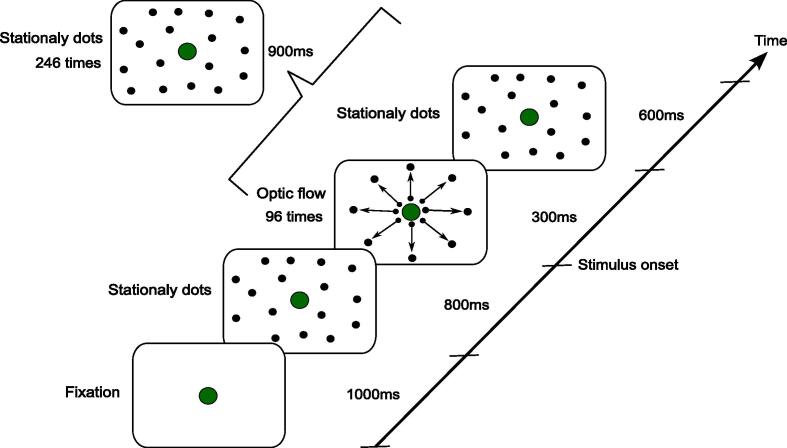
Optic-flow task paradigm. The optic-flow stimuli started with a centered fixation spot for 1,000 ms. The fixation spot was replaced with stationary dot stimuli for 800 ms. Subsequently, the dots were moved in a radial outward pattern (looming motion) on the center of the screen. After 300 ms, the stationary dots were presented for 600 ms. In the case of the stationary dot stimuli, a centered fixation spot was replaced with stationary dots for 1,700 ms. In these trials, the optic-flow stimuli occurred 96 times, and the stationary dot stimuli occurred 264 times.

The experimental paradigm of the study has been described in previous work (Noguchi-Shinohara et al., 2021). Visual stimuli were presented on the screen in front of a participant. Participants were required to maintain their centered visual fixation throughout the presentation of all the visual stimuli. Two types of stimuli, namely, stationary dots and optic-flow stimuli, were used. The stationary dot stimuli consisted of 1,000 white dots randomly presented on a black background. The optic-flow stimuli consisted of animated sequences of the 1,000 white dots moving radially outward with an average dot speed of 15°/s. This optic-flow task consisted of a total of 360 trials (120 trials × 3 runs with intervals of tens of seconds between the runs). The 360 trials were composed of 96 (32 × 3) optic-flow stimuli and 264 (88 × 3) stationary dot stimuli. Each trial had a duration of 2.7 sec for both types of stimuli. In the optic-flow stimuli, each trial started with the presentation of a fixation dot for a duration of 1,000 ms, followed by the presentation of stationary dots for 800 ms. Afterward, the presented dots moved radially outward (looming motion) from the center of the screen for 300 ms and then maintained their location for 600 ms (Fig. 1). In the stationary dot stimuli, each trial started with the presentation of a fixation dot for a duration of 1,000 ms, followed by the presentation of stationary dots for 1,700 ms (Fig. 1). It has been reported that brain activity fluctuates in response to continuous repeated visual stimuli because of long-term adaptation/habituation or fatigue (Kremlácek et al., 2007). To avoid these fluctuations in neural responses to optic-flow stimuli, we inserted stationary dot stimuli into the optic-flow task, setting the optic-flow and stationary dot stimuli to occur randomly at a ratio of 4:11. We used a single random seed to make the sequence of the stimuli, and the order of the optic-flow and stationary dot stimuli were identical among all the participants.

### 2.3 MEG data collection and analyses

#### 2.3.1 Data acquisition during the optic-flow task

The MEG measurements were performed using a MEG system (Ricoh Company, Ltd., Kanazawa, Japan) consisting of a 160-channel whole-head coaxial gradiometer. We sampled the data with a 1 kHz sampling rate, applying a 0.16 Hz high-pass filter and a 200 Hz low-pass filter for preventing the signal aliasing (anti-alias filters implemented by the instrument vendor). The position of the head within the helmet was determined by magnetic marker coils attached at five locations on the surface of the head as fiduciary points to the landmarks. The MEG was recorded with the subjects relaxed while lying supine in a magnetically shielded room. The faces and upper bodies of the participants were tracked using a video camera to monitor their wakefulness. Participants was allowed to take a break during the intervals between the task sessions to maintain the their concentration.

#### 2.3.2 Preprocessing

MEG data from 96 optic-flow trials were analyzed for each subject. The acquired sensor signals were first digitally processed using bandpass filters (1–110 Hz; 4th order butterworth), and eye movement and cardiac artifacts were removed using Preconditioned ICA for Real Data (Picard) algorithm (Ablin et al., 2018). To remove line noise (60 Hz), we applied a noise filtering method, Zapline (de Cheveigné, 2020). The stimulus onset in each trial, which was identified with a measured trigger signal, was defined as the start time of the dots moving in the optic-flow stimuli, and each epoch of interest was set from –300 to 1,000 ms. Among the 96 epochs for each subject, the epochs with muscle artifacts were removed (average 1.7 epochs per subject) using an automatic artifact rejection tool implemented in the Fieldtrip toolbox (Oostenveld et al., 2011). The remaining cleaned epochs were averaged for each subject to increase the signal-to-noise ratio.

#### 2.3.3 Source reconstruction

Isotropic voxels (5 mm) were generated in a brain region on a template MRI, resulting in 15,448 voxels within the brain region. The generated voxels were spatially warped into an individual brain, and subject-specific magnetic lead field vectors were computed for each voxel with a single-shell model approximation (Nolte, 2003). The voxels for each subject were indexed to 48 cortical/subcortical module-level regions defined in the Brainnetome atlas (Fan et al., 2016).

An array-gain constraint minimum-norm spatial filter with recursively updated gram matrix (AGMN-RUG) (Kumihashi and Sekihara, 2010) was applied to the epoch-averaged signals to obtain source power activities for all the voxels, i.e., voxel-level source time courses on 5-mm volumetric grids in the whole brain. In the AGMN-RUG source reconstruction technique, lead-field vectors are normalized; thus, center-of-the-head artifacts are avoided. Moreover, the recursive updating of the gram matrix provides a small source location bias and is robust to source correlation, which allows us to distinguish the resulting source power activities of adjacent regions in the dorsal stream.

#### 2.3.4 Normalized power and task-related activity

Voxel-level power time courses were computed by squaring the voxel-level source time courses at each timepoint. Based on the power time courses, we quantified brain activity related to the optic-flow task. Specifically, task-related brain activity was evaluated by normalizing the time-resolved power to the averaged power (baseline power) within the prestimulus interval in each epoch (the interval ranged from −300 to 0 ms, in which the stationary dot pattern was presented). We also introduced regional normalized power for the 48 regions. The regional normalized power time courses were computed by averaging the normalized voxel-level power time courses within each region.

As shown in the Results section, the normalized power time courses averaged over the whole brain for each subject had a peak with a maximum value between 100 and 200 ms after stimulus onset. Based on this observation, we considered a normalized power value at the peak time as task-related activity, that is, a representative brain response to the optic-flow stimuli, for each subject.

### 2.4 MRI acquisition and analyses

Three-dimensional T1-weighted MR images were acquired for each subject using the Sigma Excite HD 1.5 T system (GE Yokogawa Medical Systems Ltd., Milwaukee, WI, USA). The acquired MRI data were used to generate the head model for source reconstructions of the MEG sensor data and to evaluate gray matter (GM) volumes. Each MRI scan was performed on the same day as the MEG evaluation. The scan was performed with spherical lipid markers placed at the five fiducial points on which MEG marker coils were placed for co-registration of the MEG coordinate system with the MRI coordinate system. MRI images consisted of 152–158 sequential horizontal slices of 1.2 mm thickness, with a resolution of 512 × 512 points in a field of view of 261 × 261 mm. Regional GM volumes corresponding to the 48 anatomical regions (Fan et al., 2016) were computed using the Computational Anatomy Toolbox [CAT12 version 12.8.1 (1987)] (Gaser et al., 2022). The regional GM volumes were evaluated by the morphometry pipeline implemented in CAT12 with default parameters. The total intracranial volume (TIV), the sum of all segments classified as gray and white matter and cerebrospinal fluid, was also calculated for each subject. The volume of the PHG was defined as the sum of the volumes of the left- and right-hemisphere PHGs. We applied a general linear model (GLM) to the volume of the PHG to adjust for TIV and age.

### 2.5 Amyloid PET

#### 2.5.1 Data acquisition

Twenty-five of the 45 participants underwent an ^11^C-PiB-PET scan (Discovery PET/CT 600 Motion, GE Healthcare, Milwaukee, WI) at the Public Central Hospital of Matto Ishikawa to measure the Aβ burden. Five minutes of data acquisition was repeated four times starting 50 min after intravenous injection of 555 MBq ^11^C-PiB, using dynamic list-mode acquisition method. All images were reconstructed using a 3-dimensional ordered subset-expectation maximization (3D-OSEM) algorithm and Gaussian filter (a 4.0-mm full-width at half-maximum isotropic Gaussian kernel). The imaging data were acquired in 128 × 128 matrices with a pixel size of 2.0 mm and a slice thickness of 3.27 mm. A conventional computed tomography (CT) attenuation correction method was adopted.

#### 2.5.2 Quantification of amyloid-β burden

We used the global cortical standardized uptake value ratio (SUVr) as a measure of Aβ burden. The computation of the global amyloid burden was performed following the processing method described in Klunk et al. (Klunk et al., 2015). First, each individual MR image was coregistered with the Montreal Neurological Institute (MNI) template (‘avg152.T1.nii’), and each individual PET image was coregistered with the individual MR image. Next, we applied segmentation to individual MR images and normalized individual PET and MR images in the MNI space. PiB SUVs were then extracted from the normalized PET images using standard volume-of-interest (VOI) templates for the cortical region (‘voi_ctx_2mm.nii’) and the whole cerebellum (WC) (‘voi_WhlCbl_2mm.nii’) with the generation of isotropic voxels (2 mm) in the MNI template. VOI templates were downloaded from the Global Alzheimer’s Association Interactive Network (GAAIN) website (https://www.gaain.org/centiloid-project). The SUVr was calculated as the ratio of the SUV in the global cortical target region to that in the WC. We applied a general linear model (GLM) to the global amyloid burden (SUVr) that was adjusted for age and the difference between PET and MEG dates.

### 2.6 Statistical analyses

To examine which brain regions are most activated to the optic-flow task, baseline power (before stimulus onset) and task-induced peak power (after stimulus onset) for all 48 regions were evaluated using one-sample t-tests (*p* < 0.05). The false-discovery-rate (FDR) adjusted *p*-value (i.e., *q*-value) below 0.05 was considered statistically significant.

To evaluate the correlation between global amyloid burden and PHG volume with task-related activity at each region, we performed correlation coefficient analysis on 25 participants who underwent PET scans. The normality of the task-related activity (the normalized power at a peak) was assessed using the Shapiro–Wilk test. Regional task-related activities had a skewed distribution; therefore, we used logarithmic transformation values (common logarithms) of task-related activity in the statistical analyses. The distribution of the transformed data was approximately normal, allowing us to perform correlation analyses with parametric statistics (the kurtosis and skewness values of the distributions for each region are shown in Supplementary Table 1). SUVr values of the amyloid burden were adjusted for age and the difference between PET and MEG dates before correlation analysis. In addition, the PHG volume was adjusted for age and TIV. Correlation analyses with regional task-related activities were performed using the adjusted values. We did not adjust gender differences in correlation analyses because there was no difference between males and females in amyloid deposition in our sample (male: Mean ± SD = 1.63 ± 0.39, female; Mean ± SD = 1.66 ± 0.41, *p* = 0.835). We considered *p* < 0.05 to indicate statistical significance in the correlation analyses.

## 3 Results

### 3.1 Participant characteristics

This study included a total of 45 participants, consisting of 8 patients with AD dementia, 16 patients with MCI, and 21 CU participants. The CDR scores were 0 for the CU participants (n = 21), 0.5 for patients with MCI (n = 16), and 1 (n = 7) or 2 (n = 1) for patients with AD dementia. The typical response to the optic-flow task was investigated in the 21 CU participants (10 females, 11 males; mean age ± SD, 73.1 ± 5.0 years; range 66–83 years; mean MMSE score ± SD, 29.1 ± 1.1). Twenty-five of 45 participants underwent an ^11^C-PiB-PET scan to measure the Aβ burden. We analyzed the association of task-related activities with global amyloid burden and PHG volume in this subgroup of 25 subjects. The subgroup included 24 AD patients [CDR: 0.5 (n = 16), 1 (n = 7), and 2 (n = 1)] and one CU participant [CDR 0 (n = 1)] (13 females, 12 males; mean age ± SD, 72.3 ± 9.4 years; range 53–85 years; mean MMSE score ± SD, 23.4 ± 4.8).

### 3.2 Identification of task-related activities

**Fig. 2:**
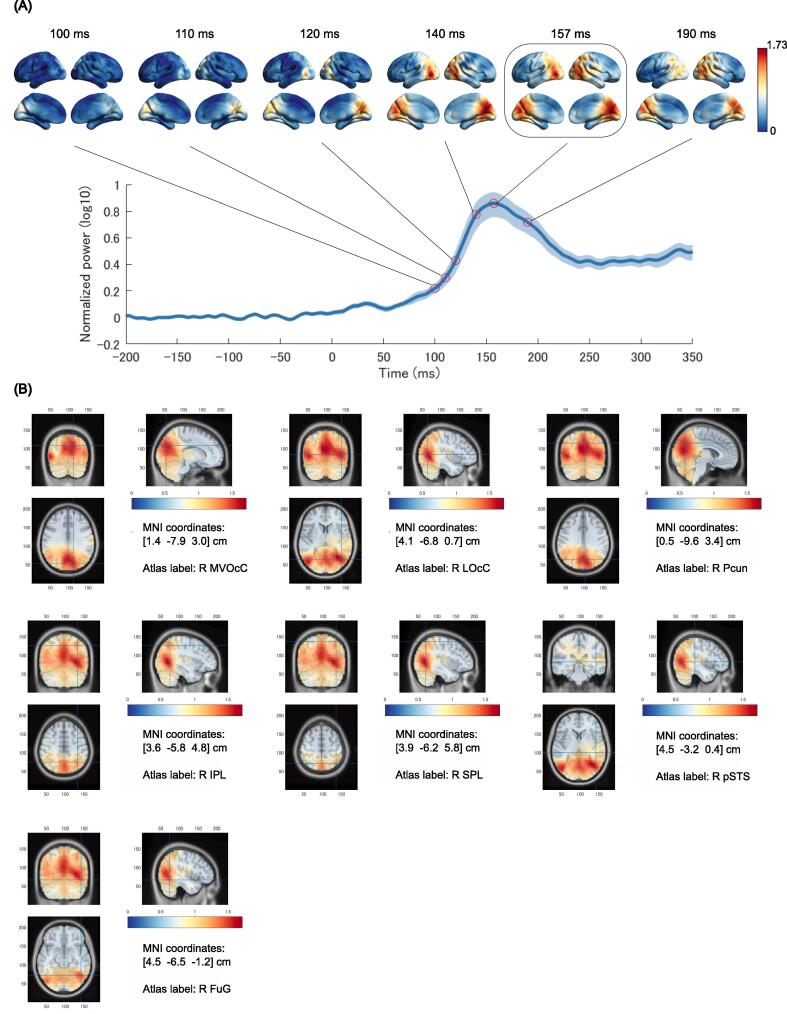
Regional brain activities as a response to optic-flow stimuli. (A) (*Upper*) Overall average of the brain activations in the source space at 100, 110, 120, 140, 157, and 190 ms. (*Lower*) Overall average of voxel-time courses in the whole brain (normalized to baseline). The maximum peak was located at 157 ms after optic-flow stimulus onset. The data are presented as the means ± SEs. (B) MEG source localization of task-evoked activity in the MVOcC, LOcC, Pcun, IPL, SPL, pSTS, and FuG at 157 ms.

Representative task-related regions where large brain activities occurred in response to optic-flow stimuli were identified by computing the normalized power time courses from the MEG data for the 21 CU subjects (Fig. 2). As shown in Fig. 2A, the normalized power of the whole brain was computed by averaging the normalized power over the whole brain for the 21 subjects. For all subjects, peaks in the normalized power of the whole brain were observed between 100 and 200 ms after stimulus onset. On average, the maximum normalized power was observed at 157 ms. The activation occurred in turn from the occipital lobe to the parietal lobe (upper row in Fig. 2A; Supplementary Fig. 1). We also evaluated the peak regional normalized power by averaging across voxels in each region and then identified the regions where the peak power had a large value using the modular-level 48 Brainnetome atlas. Top largest power values were observed at 157 ms in the left and right medial ventral occipital cortices (MVOcCs), lateral occipital cortices (LOcCs, V5/hMT + ), Pcun, IPLs, superior parietal lobules (SPLs), posterior superior temporal sulcus (pSTS), and fusiform (FuG) in CU subjects (Supplementary Table 2; the largest power value was observed in R Pcun.). One-sample *t*-test showed significant differences in all brain regions (*q*-value < 0.05), indicating that the brain responded in some way overall to the optic-flow stimulus. From the 48 regions, we selected the top 14 regions (7 regions on each side) with the highest power values, which includes the dorsal pathway, as ROIs: bilateral MVOcCs, LOcCs, Pcuns, IPLs, SPLs, pSTS, and FuG (Fig. 2B). The values of the peak power in the 14 ROIs were considered in the subsequent correlation analyses of task-related activity in each region with global amyloid burden and PHG volume.

We also performed group comparisons between the AD/MCI group (n = 24) and the CU group (n = 21) regarding task-related brain activity across the 14 ROIs using *t*-tests (Supplementary Table 3). Apart from the right pSTS, there was a tendency for brain activity to be lower in the AD group compared to the CU group across all other regions, but there were no statistically significant differences between them across all the 14 regions.

### 3.3 Global amyloid burden is negatively correlated with task-related activity

**Fig. 3:**
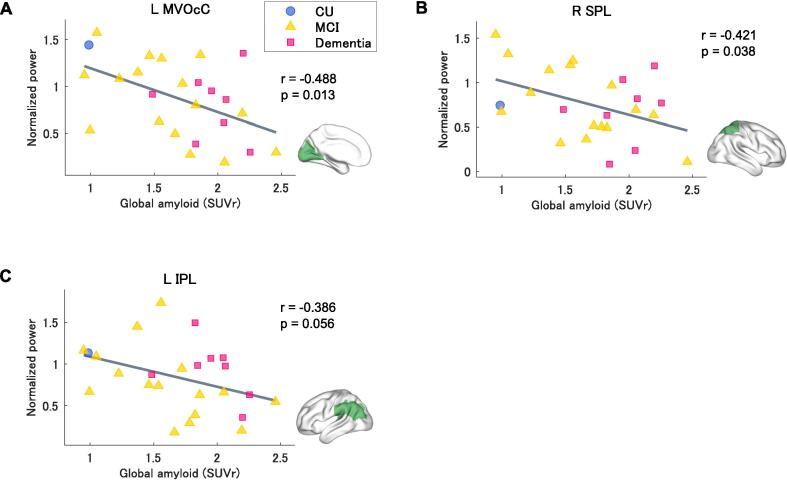
Correlation analyses of the association between regional brain activity and global amyloid burden (SUVr) in 25 participants. The dots represent the data for each subject. Blue: control (CDR 0), orange: MCI (CDR 0.5), and pink: mild or moderate AD dementia (CDR 1 or 2). The green areas represent the brain regions that were selected for the correlation analysis. (For interpretation of the references to colour in this figure legend, the reader is referred to the web version of this article.)

Fig. 3 shows the correlation analyses of the association between regional brain activity and global amyloid burden in 25 participants. Global amyloid burden was negatively correlated with task-related activity in the left MVOcC and right SPL (*r* = -0.488, *p* = 0.013 and *r* = -0.421, *p* = 0.038, respectively) (Fig. 3A and 3B). A trend toward a negative correlation was also observed between the global amyloid burden and task-related activity in the left IPL (*r* = -0.386, *p* = 0.056) (Fig. 3C). There were no significant correlations between global amyloid burden and task-related activity in the other ROIs (Supplementary Fig. 2). Supplementary Fig. 2 shows the results of each analysis in all ROIs.

### 3.4 PHG volume is positively correlated with task-related activity

**Fig. 4:**
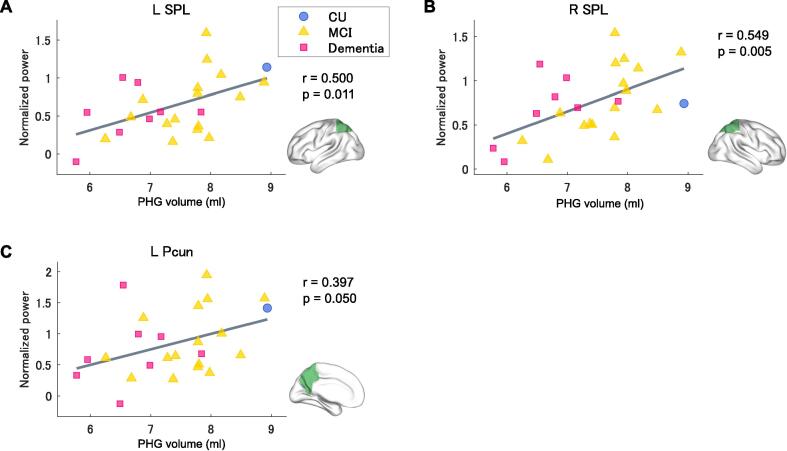
Correlation analyses of the association between regional brain activity and PHG volume for 25 participants. The dots represent the data for each subject. Blue: control (CDR 0), orange: MCI (CDR 0.5), and pink: mild or moderate AD dementia (CDR 1 or 2). The green areas represent the brain regions that were selected for the correlation analysis. (For interpretation of the references to colour in this figure legend, the reader is referred to the web version of this article.)

The results of the correlation analysis between PHG volume and task-related activity in each ROI are shown in Fig. 4. A significant positive correlation was observed between PHG volume and task-related activity in the left and right SPL (*r* = 0.500, *p* = 0.011 and *r* = 0.549, *p* = 0.005, respectively) (Fig. 4A and 4B). A trend toward a positive correlation was also observed between PHG volume and task-related activity in the left Pcun (*r* = 0.397, *p* = 0.050) (Fig. 4C). There were no significant correlations between PHG volume and task-related activity in the other ROIs (Supplementary Fig. 3). Supplementary Fig. 3 shows the results of each analysis in all ROIs.

## 4 Discussion

The term “visuospatial” comprises ‘visual’ and ‘spatial’. Visuospatial perception refers to the ability to process and interpret visual information about spatial locations of objects. Visuospatial information is thought to be processed by a dorsal stream occipito-parietal pathway. In the present study, we investigated the regional distributions of task-related neural activity evoked by an optic-flow stimulus and its correlation with AD progression markers. MEG recordings demonstrated that the brain response to the optic-flow task indeed occurred mainly in the dorsal stream. The important findings of our study were that the amyloid burden and PHG volume were correlated with task-related activity in the SPL and MVOcC, that task-related activities in the left MVOcC and right SPL were negatively correlated with amyloid burden, and that activities in the bilateral SPL were positively correlated with PHG volume. There were no significant correlations for the other ROIs for either amyloid burden or PHG volume; however, there was a tendency for greater AD pathology to be associated with lower task-related activity. Since the SPL is known to be responsible for visuospatial perception, our results indicate that the altered brain activity evoked by the optic-flow task may indicate the influence of visuospatial impairment associated with AD.

The identification of task-related activities in the 21 CU subjects provided the first MEG evidence that in healthy elderly participants, activations related to the optic-flow task are routed from the occipital cortex to the occipito-parietal regions via the MT+/V5 and V6 regions. The five bilateral areas configuring the entire dorsal stream were involved in the response, similar to previous MEG (Holliday and Meese, 2008), PET (de Jong et al., 1994, Ptito et al., 2001) and fMRI (Cardin and Smith, 2010, de Jong et al., 1994, Pitzalis et al., 2015, Smith et al., 2006, Sunaert et al., 1999, Uesaki and Ashida, 2015) studies of young healthy subjects.

It has been reported that the SPL is a key area in visuospatial perception (Breveglieri et al., 2021, Culham and Kanwisher, 2001, Prvulovic et al., 2002, Sack, 2009, Ward et al., 2016). A reduction in optic-flow-task-related activities in the SPL may therefore be caused by visuospatial impairment in AD patients. This finding is consistent with several previous fMRI studies reporting that visuospatial impairment in AD patients was associated with the SPL (Prvulovic et al., 2002, Thulborn et al., 2000, Vannini et al., 2007).

The laterality of the SPL in our study indicates that visuospatial functions are predominantly attributed to the right parietal lobules (Ciçek et al., 2009, Fierro et al., 2000, Fink et al., 2000). A previous MEG study demonstrated that brain activities evoked by optic-flow stimuli were more prominent in the right hemisphere than in the left hemisphere (Holliday and Meese, 2008). This finding indicates that the response to optic-flow stimuli is more sensitive in the right SPL, which is consistent with the finding in our study that a correlation between task-related activity and the global Aβ burden was found only in the right SPL. The associations of task-related activity in the right SPL with both PHG volume loss and global Aβ burden may indicate functional changes due to the death of neural cells as well as synaptic dysfunction caused by the accumulation of Aβ, which leads to a reduction in optic-flow task-related activities. This consideration of the causes of functional changes is consistent with a previous report that cognitively healthy individuals showed fewer signs of brain volume loss, whereas MCI/AD patients showed brain atrophy (Basu et al., 2023).

The MVOcC, composed of the lingual gyrus and cuneus, is necessary for both basic and higher-level visual processing. More broadly, the cuneus and lingual gyri house functional areas V1 to V4 and facilitate proper functioning of the ventral and dorsal streams (Baker et al., 2018, Cornette et al., 1998). These two gyri have been implicated in both the basic and higher-order visual processing required for the direction and speed of visual motion (Grill-Spector and Malach, 2004, Orban et al., 1998). In a behavioral test study, it was shown that AD patients exhibit impaired processing of the radial motion patterns of optic flow, which leads to an individual’s sense of self-motion (O’Brien et al., 2001, Tetewsky and Duffy, 1999). Visual cortical areas appear to be vulnerable to AD because of a significant amount of cell loss, particularly in some layers of the primary and secondary visual cortices (Sartucci et al., 2010). Because these cells have long cortical projections to the MT, neuron loss in AD patients disrupts the transmission of visual signals from V1 to the MT, causing deficits in higher-order visual processing (Hof, 1994). Thus, our results suggest that altered neuronal activity in the MVOcC, that is, the disruption of long projections from V1 to the MT by the loss of cells in the MVOcC, may contribute to the brain activity response to the optic-flow task.

The present study also showed that there was a tendency for the task-related brain activity to be lower in the AD group compared to the CU group in most of the ROIs although the group differences were not statistically significant. This result suggests that brain responses to the optic-flow task gradually decrease as AD progresses. Considering the results of the correlation analysis, these findings suggest that the response to the optic-flow task does not abruptly deteriorate due to cognitive decline, but rather decreases gradually over time.

Recently, it has been reported that posterior cortical atrophy (PCA) could be a predictive syndrome for AD neuropathological features (Chapleau et al., 2024). Patients with PCA typically have normal cognition at early stages but show visuospatial impairment unlike classic memory loss symptoms, and therefore they often face a delay in diagnosis. Optic-flow task via MEG shown in this study might be useful for early detection of PCA and stratification of AD at early stage.

There are several limitations in our study. First, our results did not show whether the optic-flow task can be used to predict the gradation of the disease process. AD is heterogeneous in nature. A recent study reported four distinct subtypes of AD, including bilateral parietal, frontal, and temporal atrophy AD subtypes (occipital sparing AD (OSAD), 29.2 %; left temporal dominant atrophy AD (LTAD), 22.4 %; minimal atrophy AD (MAD), 16.1 %; and diffuse atrophy AD (DAD), 32.3 %) (Zhang et al., 2021). Future efforts to characterize MEG activity in AD subtypes and in other pathologies are necessary. Second, the associations among Aβ pathology, PHG atrophy, and optic-flow task performance reported in this study were cross-sectional. The causal relationships between these biomarkers of AD pathology and visuospatial perception decline require longitudinal investigation.

## 5 Conclusion

The present results suggested that AD causes alterations in visuospatial perception in the SPL and MVOcC during optic-flow stimuli with looming motion and that MEG during the optic-flow task captures changes in the brain activity associated with visuospatial impairment caused by AD.

## CRediT authorship contribution statement

**Shoko Tsuchimine:** Writing – original draft, Validation, Investigation, Formal analysis, Data curation. **Kiwamu Kudo:** Writing – review & editing, Supervision, Software, Methodology, Formal analysis, Conceptualization. **Junji Komatsu:** Data curation. **Shutaro Shibata:** Data curation. **Sachiko Kitagawa:** Data curation. **Yoshihiro Misaka:** Writing – review & editing, Supervision. **Moeko Noguchi-Shinohara:** Writing – review & editing, Supervision, Project administration. **Kenjiro Ono:** Project administration. **Hirofumi Morise:** Writing – review & editing, Supervision, Project administration, Methodology, Formal analysis. **Takashi Asakawa:** Writing – review & editing, Supervision, Project administration.

## Declaration of competing interest

The authors declare the following financial interests/personal relationships which may be considered as potential competing interests: ST, KK, HM, YM and TA are employees of Ricoh Company, Ltd. The authors declare that no other competing interests exist. The other authors have no competing financial conflicts of interest to disclose.

## Data Availability

The authors do not have permission to share data.
