# Supplementary material for: Magnetoencephalographic brain activity evoked by the optic-flow task is correlated with β-amyloid burden and parahippocampal atrophy
Source: Neuroimage Clin. 2024 Nov 4;44:103700. doi: 10.1016/j.nicl.2024.103700 (PMC11585792; doi:10.1016/j.nicl.2024.103700)
Supplement: Supplementary Data 1 [file mmc1.docx]

| Regions |  | Raw data | | |  | Transformed data (log10) | | |
| --- | --- | --- | --- | --- | --- | --- | --- | --- |
|  |  | skewness | kurtosis | *p*-value |  | skewness | kurtosis | *p*-value |
| L FuG |  | 5.498 | 2.145 | <0.001 |  | 0.488 | -0.466 | 0.915 |
| L pSTS |  | 0.965 | 1.495 | <0.001 |  | -1.093 | 0.275 | 0.244 |
| L SPL |  | -0.784 | 0.805 | 0.006 |  | -1.275 | -0.097 | 0.246 |
| L IPL |  | 24.716 | 5.595 | 0.002 |  | 0.559 | -.645 | 0.443 |
| L Pcun |  | 1.587 | 1.627 | <0.001 |  | -0.527 | -0.107 | 0.810 |
| L MVOcC |  | 7.635 | 2.608 | <0.001 |  | -0.512 | 0.342 | 0.877 |
| L LOcC |  | 3.554 | 2.050 | <0.001 |  | -0.192 | 0.226 | 0.689 |
| R FuG |  | 10.990 | 3.175 | <0.001 |  | -0.072 | 0.608 | 0.263 |
| R pSTS |  | 10.804 | 3.116 | <0.001 |  | 0.467 | -0.301 | 0.606 |
| R SPL |  | 1.079 | 1.137 | 0.024 |  | -0.968 | -0.416 | 0.244 |
| R IPL |  | 1.884 | 1.393 | 0.009 |  | -0.671 | -0.141 | 0.858 |
| R Pcun |  | 8.938 | 2.874 | <0.001 |  | -0.163 | 0.551 | 0.740 |
| R MVOcC |  | 5.200 | 2.390 | <0.001 |  | -0.128 | 0.873 | 0.048 |
| R LOcC |  | 1.854 | 1.519 | <0.001 |  | -1.060 | 0.154 | 0.606 |

Supplementary Table 1

Normal distribution test using the Shapiro-Wilk test for raw and transformed task-related activity data.

Skewness is a measure of symmetry. A distribution with zero skewness is perfectly symmetrical, meaning the left and right sides of the distribution are mirror images. Kurtosis is a measure of whether the data distributions are heavy-tailed or light-tailed relative to a normal distribution. A p-value that is smaller than the level of risk (< 0.05) indicates an observed sample that is not sufficiently normal distribution. Regional task-related activities of raw data had a skewed distribution, therefore we used logarithmic transformation values. The distribution of the transformed data was approximately normal.

| Rank | Region | mean power |  | p-value |  | q-value |
| --- | --- | --- | --- | --- | --- | --- |
| 1 | R Pcun | 28.68692908 |  | 0.010899 |  | 0.010899 |
| 2 | R MVOcC | 26.78317836 |  | 0.006152 |  | 0.006562 |
| 3 | L Pcun | 24.84670358 |  | 0.000869 |  | 0.00106 |
| 4 | R LOcC | 22.47294056 |  | 0.000165 |  | 0.000273 |
| 5 | L MVOcC | 20.55282435 |  | 0.00168 |  | 0.001875 |
| 6 | L LOcC | 17.55635038 |  | 0.000487 |  | 0.000668 |
| 7 | R IPL | 13.05760751 |  | 1.14E-05 |  | 4.99E-05 |
| 8 | R SPL | 12.86488296 |  | 3.94E-05 |  | 9.96E-05 |
| 9 | L IPL | 12.09455067 |  | 2.32E-05 |  | 7.12E-05 |
| 10 | R FuG | 11.60185067 |  | 0.009799 |  | 0.010007 |
| 11 | L FuG | 10.79936884 |  | 0.000539 |  | 0.000718 |
| 12 | L SPL | 10.35525443 |  | 2.29E-05 |  | 7.12E-05 |
| 13 | R pSTS | 9.078496064 |  | 0.008002 |  | 0.00835 |
| 14 | L pSTS | 8.162751133 |  | 0.001444 |  | 0.00165 |
| 15 | R CG | 7.863315156 |  | 0.000271 |  | 0.000407 |
| 16 | L CG | 7.613608106 |  | 4.4E-06 |  | 3.81E-05 |
| 17 | L ITG | 7.518552805 |  | 4.78E-05 |  | 0.000115 |
| 18 | R Tha | 6.966082455 |  | 0.000141 |  | 0.00025 |
| 19 | L MTG | 6.54253549 |  | 7.61E-05 |  | 0.000152 |
| 20 | R ITG | 6.445855162 |  | 0.000798 |  | 0.001008 |
| 21 | R MTG | 6.266519265 |  | 2.85E-05 |  | 8.04E-05 |
| 22 | L Tha | 6.262529689 |  | 0.000728 |  | 0.000944 |
| 23 | R PoG | 5.974623061 |  | 0.000119 |  | 0.000229 |
| 24 | L PoG | 5.246643926 |  | 2.37E-05 |  | 7.12E-05 |
| 25 | L Hipp | 5.171329525 |  | 0.00014 |  | 0.00025 |
| 26 | R INS | 5.100734337 |  | 0.000361 |  | 0.000526 |
| 27 | R Hipp | 5.041752376 |  | 0.000184 |  | 0.000295 |
| 28 | R STG | 4.880903124 |  | 6.43E-05 |  | 0.00014 |
| 29 | L PCL | 4.733040825 |  | 6.71E-05 |  | 0.00014 |
| 30 | R BG | 4.60535558 |  | 6.23E-05 |  | 0.00014 |
| 31 | R PhG | 4.591414803 |  | 0.000884 |  | 0.00106 |
| 32 | L PhG | 4.334375934 |  | 3.29E-05 |  | 8.79E-05 |
| 33 | R PrG | 4.252886231 |  | 3.51E-06 |  | 3.81E-05 |
| 34 | R PCL | 4.20853734 |  | 7.09E-06 |  | 3.81E-05 |
| 35 | L PrG | 4.093173242 |  | 2.17E-05 |  | 7.12E-05 |
| 36 | R IFG | 3.784710032 |  | 7.29E-06 |  | 3.81E-05 |
| 37 | L BG | 3.540796077 |  | 0.000251 |  | 0.000389 |
| 38 | R Amyg | 3.418921778 |  | 0.002539 |  | 0.00277 |
| 39 | L STG | 3.36312398 |  | 0.00016 |  | 0.000273 |
| 40 | R MFG | 3.269094449 |  | 4.78E-06 |  | 3.81E-05 |
| 41 | L INS | 3.245120729 |  | 0.000477 |  | 0.000668 |
| 42 | R OrG | 3.072946976 |  | 7.95E-06 |  | 3.81E-05 |
| 43 | L Amyg | 2.707794108 |  | 0.001121 |  | 0.001313 |
| 44 | L OrG | 2.574223103 |  | 6.19E-06 |  | 3.81E-05 |
| 45 | R SFG | 2.568139547 |  | 8.89E-07 |  | 2.13E-05 |
| 46 | L MFG | 2.553891197 |  | 2.61E-06 |  | 3.81E-05 |
| 47 | L SFG | 2.549606356 |  | 8.48E-08 |  | 4.07E-06 |
| 48 | L IFG | 2.354491192 |  | 1.64E-05 |  | 6.57E-05 |

Supplementary Table 2

Ranking of all brain regions according to mean power among the 48 evaluated regions in CU subjects.

| Regions | MCI/AD (n=24) | | CU (n=21) | | *t*-value | Uncorrected *p*-value |
| --- | --- | --- | --- | --- | --- | --- |
|  | Mean | SD | Mean | SD |  |  |
| L_SPL | 0.615 | 0.404 | 0.835 | 0.433 | 1.759 | 0.086 |
| L_IPL | 0.825 | 0.405 | 0.933 | 0.335 | 0.967 | 0.339 |
| L_Pcun | 0.822 | 0.546 | 1.104 | 0.514 | 1.777 | 0.083 |
| L_MVOcC | 0.844 | 0.401 | 1.059 | 0.454 | 1.687 | 0.099 |
| L_LOcC | 0.957 | 0.398 | 1.044 | 0.413 | 0.718 | 0.477 |
| L_FuG | 0.694 | 0.336 | 0.799 | 0.437 | 0.914 | 0.366 |
| L_pSTS | 0.744 | 0.583 | 0.584 | 0.537 | -0.952 | 0.346 |
| R_SPL | 0.753 | 0.401 | 0.921 | 0.429 | 1.358 | 0.181 |
| R_IPL | 1.013 | 0.395 | 0.991 | 0.320 | -0.200 | 0.843 |
| R_Pcun | 0.869 | 0.534 | 1.108 | 0.535 | 1.501 | 0.141 |
| R_MVOcC | 0.732 | 0.404 | 1.123 | 0.482 | 2.953 | **0.005** |
| R_LOcC | 0.975 | 0.418 | 1.164 | 0.397 | 1.545 | 0.130 |
| R_FuG | 0.742 | 0.410 | 0.757 | 0.476 | 0.119 | 0.906 |
| R_pSTS | 1.028 | 0.561 | 0.625 | 0.547 | -2.431 | **0.019** |

Supplementary Table 3

Group comparisons of task-related brain activity in the 14 ROIs between the MCI/AD and CU groups. The 2-tailed Student’s *t*-test were performed for the age- and sex-adjusted values of the normalized power. Task-related brain activity in the right MVOcC and pSTS exhibited statistically significant differences between the two groups (uncorrected *p*= 0.005 and 0.019, respectively; a *p*-value < 0.05 was considered significant). However, none of the brain activity within the 14 ROIs showed statistically significant differences after multiple comparison (Bonferroni correction; a *p*-value < 0.0036(=0.05/14) was considered significant).

Supplementary Figure 1

Spatiotemporal patterns of cortical activity in the optic-flow task. Each map shows the mean value over a 5 ms time window.

Supplementary Figure 2

Correlation analyses of the association between task-related activity and global amyloid burden (SUVr) in all ROIs. The dots represent the data for each subject. Blue: control (CDR 0), orange: MCI (CDR 0.5), and pink: mild or moderate AD dementia (CDR 1 or 2). The green areas represent the brain regions that were selected for the correlation analysis.

Supplementary Figure 3

Correlation analyses of the association between task-related activity and PHG volume in all ROIs. The dots represent the data for each subject. Blue: control (CDR 0), orange: MCI (CDR 0.5), and pink: mild or moderate AD dementia (CDR 1 or 2). The green areas represent the brain regions that were selected for the correlation analysis.
