# Supplementary figures and images for: Magnetoencephalographic brain activity evoked by the optic-flow task is correlated with β-amyloid burden and parahippocampal atrophy
Source: Neuroimage Clin. 2024 Nov 4;44:103700. doi: 10.1016/j.nicl.2024.103700 (PMC11585792; doi:10.1016/j.nicl.2024.103700)

100 ms

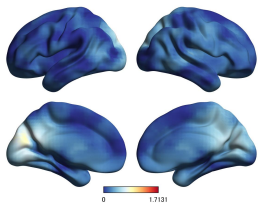

105 ms

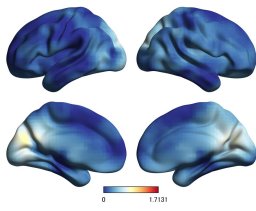

110 ms

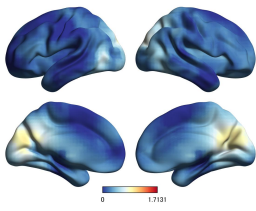

115 ms

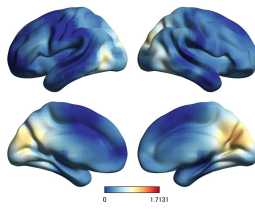

120 ms

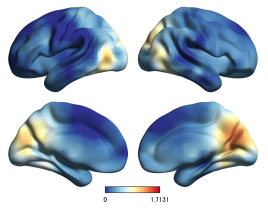

125 ms

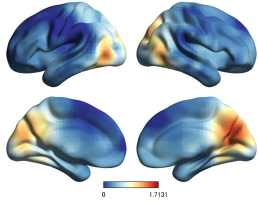

130 ms

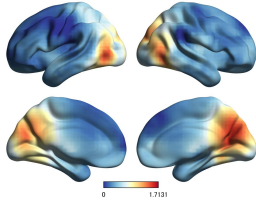

135 ms

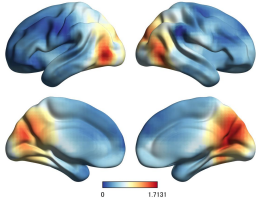

140 ms

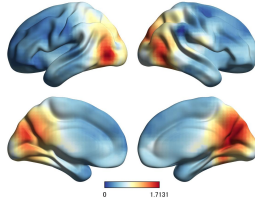

145 ms

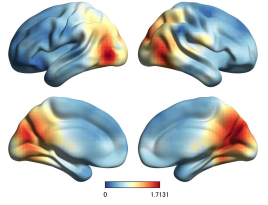

150 ms

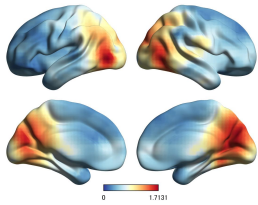

155 ms

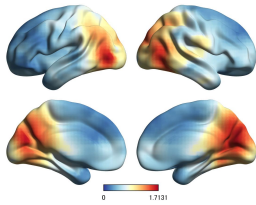

160 ms

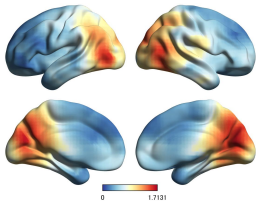

165 ms

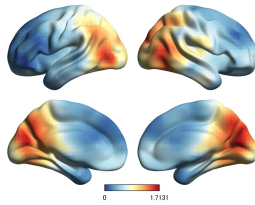

170 ms

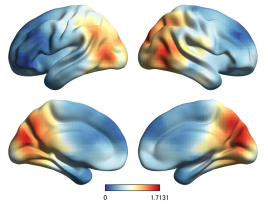

175 ms

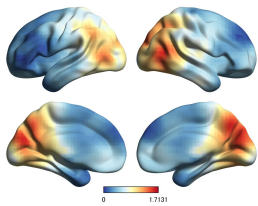

180 ms

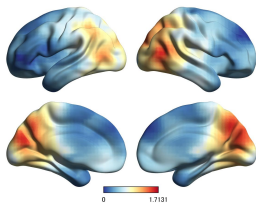

185 ms

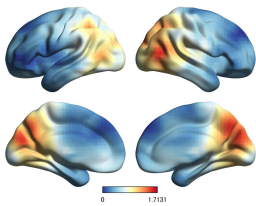

190 ms

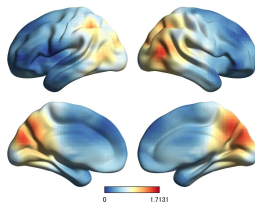

195 ms

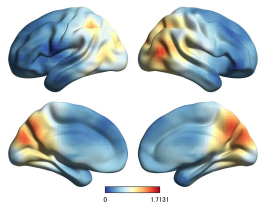

200 ms

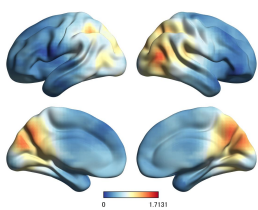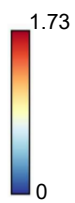

Supplement: Supplementary Data 2 [file mmc2.pdf]

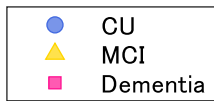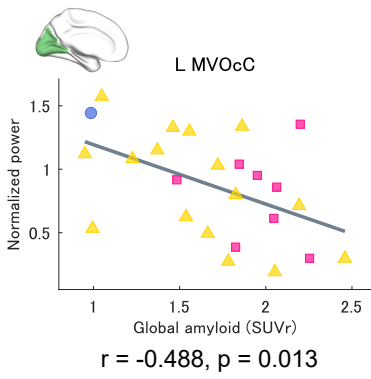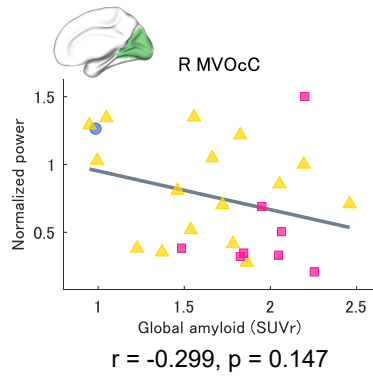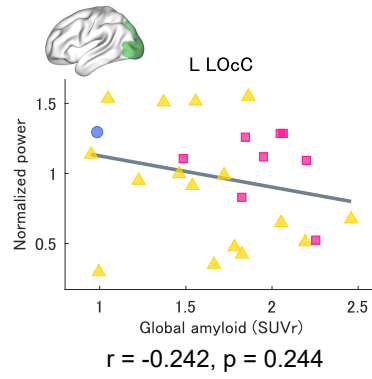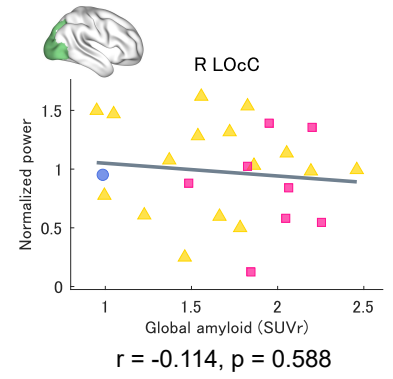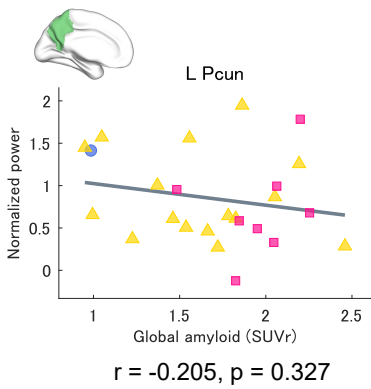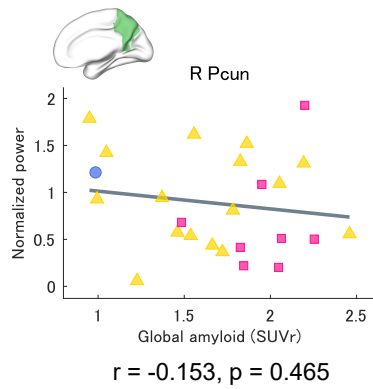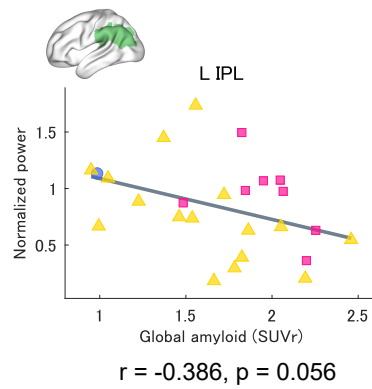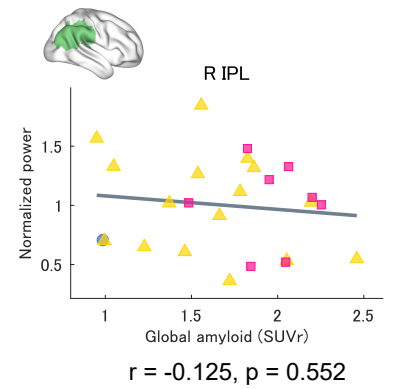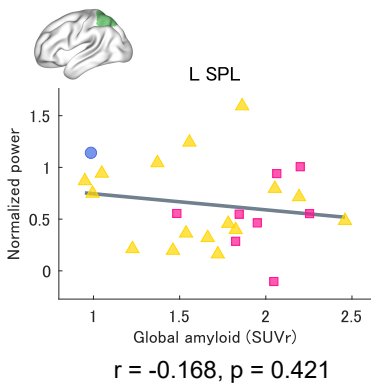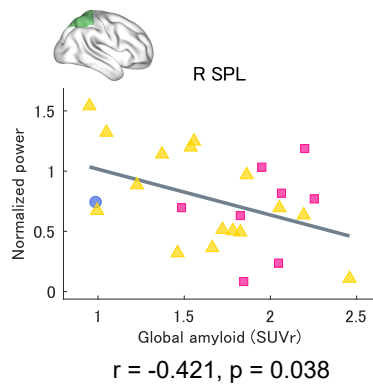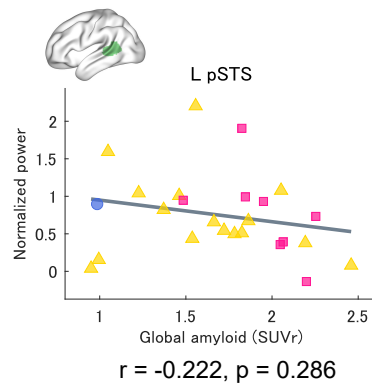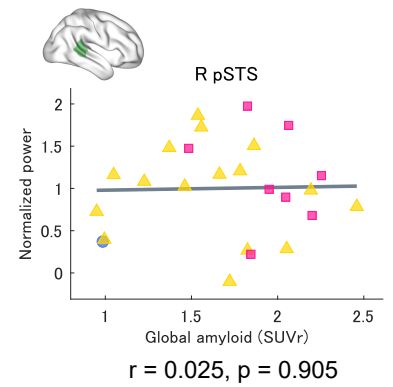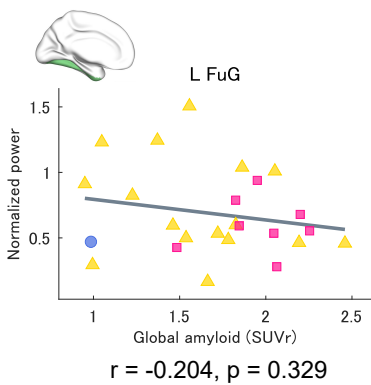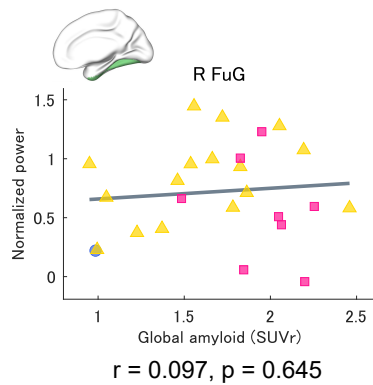

Supplement: Supplementary Data 3 [file mmc3.pdf]

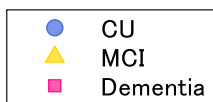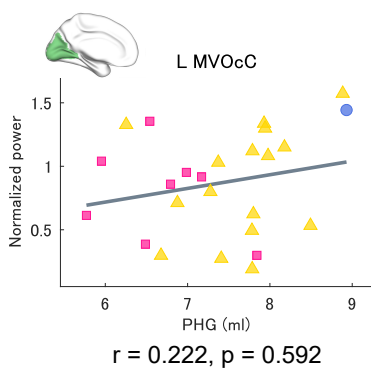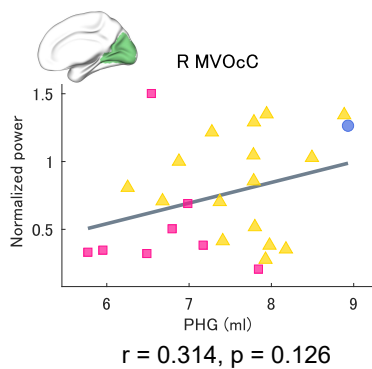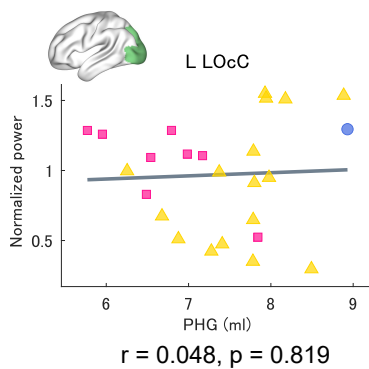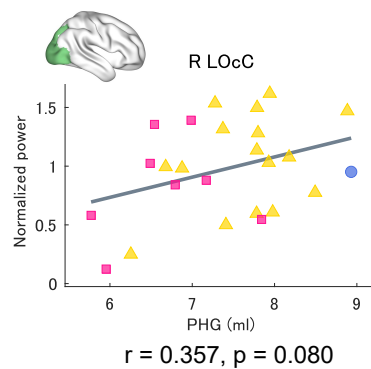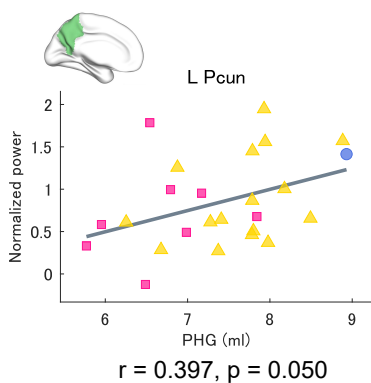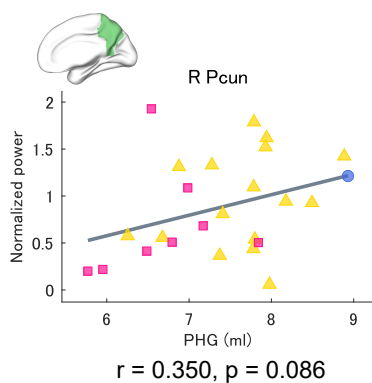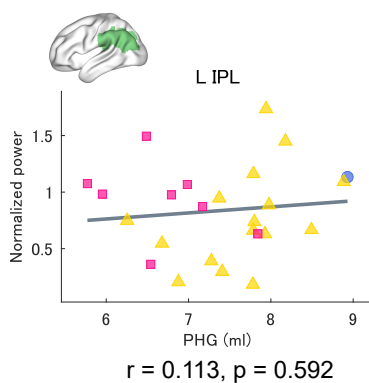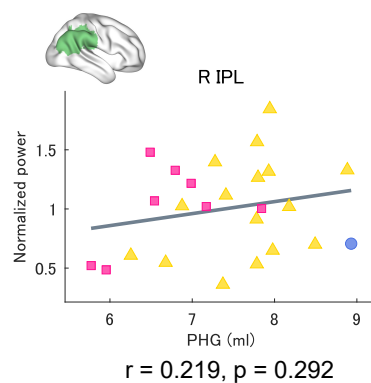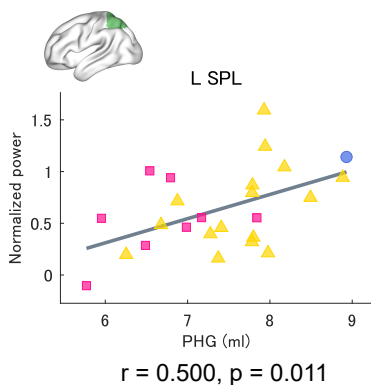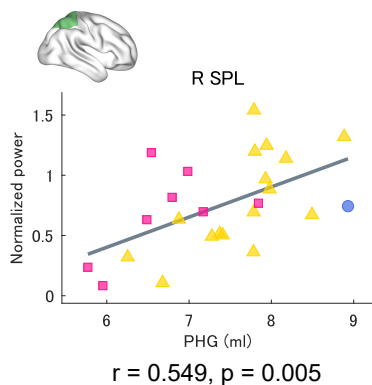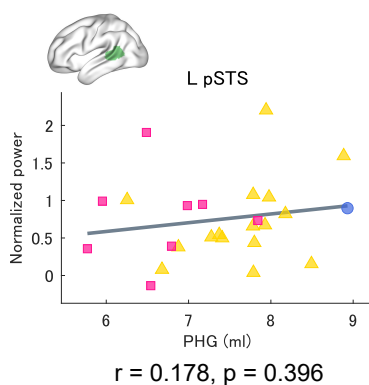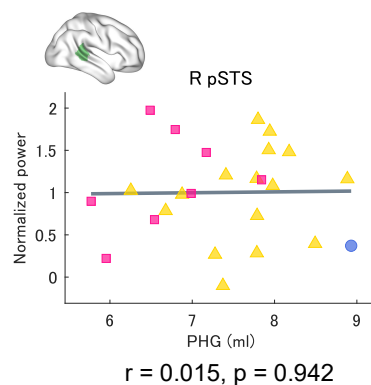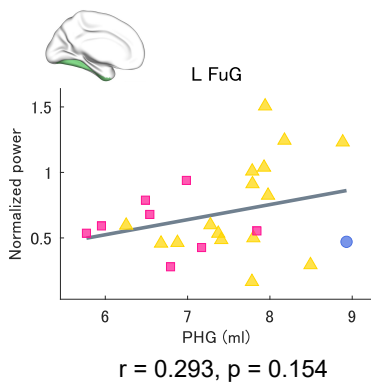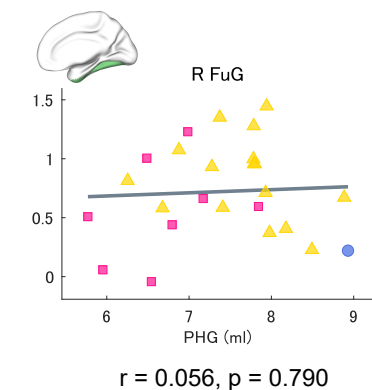

Supplement: Supplementary Data 4 [file mmc4.pdf]
